# The Great International Tuberculosis Congress

**Published:** 1904-08

**Authors:** 


*Dr. F. E. Daniel, President State Medical Association of Texas, Austin, Texas.*

### THE GREAT INTERNATIONAL TUBERCULOSIS CONGRESS.

At St. Louis, the first week in October next, there will assemble the largest, ablest and most representative body of medical and lay sanitary scientists from all parts of the civilized world that ever met anywhere.

The cause that brings them together is that great problem in sanitary science and political economy that so largely affects the welfare,—nay, the integrity of civilized peoples: *How to stay the ravages of consumption.* They will meet, not for mere academic discussion, or for clinical and pathological hair-splitting, but to devise and put in execution measures of prevention—the only potent way of dealing with the scourge. Encouraged by the success with which sanitary science has dealt with so many other destructive diseases,—the eradication of cholera, plague, diphtheria, smallpox, and now that great Southern scourge, the "saffron demon," which like a deadly upas casts its shadow annually over our land, the dreaded yellow fever, it is hoped that in this assembling of learned men some Finlay, Jenner, Koch, or Pasteur may arise; some Moses to show the way. Every European nation, some of the Asiatic, and all the American States, North and South, including, of course, the Dominion of Canada, and Mexico, will be represented by their ablest men, and a gigantic and far-reaching reform will be inaugurated. It is hoped to enlist the law-making powers of every government in the work, for, without legislation and money and earnest and capable workers, little can be done.

People, congresses and legislatures are slow to recognize that the public health is the foundation stone upon which alone a strong, vigorous and progressive nation can exist. Given healthy vigorous units and we have strong nations; and the fittest servive in nations as in individuals. But there is an awakening the world over to the necessity of successful sanitary warfare against this insidious, ever-present foe that saps the foundation sills of the fabric. Given a consumptive people, and decay and death await the State. When the Society Islands were discovered in the sixteenth century, there were 200,000 stalwart natives who lived out of doors. In twenty years consumption and smallpox, and whisky introduced by the Europeans and English, had reduced the population to 20,000. See Dr. Senn's great book on "The Island of Tahita," now being published serially.

The problem involves reform in almost every feature of civilized life; in our dwellings, factories, travel, clothing, everything.

I anticipate great enthusiasm and the shaping of means to ends that must be ultimately successful to a large degree.

It is a little disgusting to know that there is a clique of disgruntled medical men in the East who have *stooped* to misrepresent and belittle the Congress, and, by so doing,—seemingly for personal reasons,—endeavored to disaffect certain European delegates and officers of the Congress already appointed and pledged. It is that same pharisaical "I am holier than thou" crowd who sought to defeat the Ninth International Medical Congress and prevent its meeting in America in 1886; the new code soreheads who couldn't rule, sought to ruin; the dog in the manger spirit. "Since I can not prove a lover [hero], I am determined to prove a villain," as the inhuman hunch-back Duke of Gloucester said. Well, let them play the villain all they like, it will not stop the Congress. It is an assured success.

I wish I had room for a complete list of officers, but I have not. I give below the names of the Texas officers and delegates.

ADVANCE NOTES OF THE CONGRESS (from *Medico-Legal Journal*):

The government of the United States has furnished the management of the Congress copies of letters sent by Lord Lansdowne, British foreign office, through Ambassador Hon. Joseph H. Choate, announcing the acceptance of the invitation by the Dominion of Canada and from several of the Spanish-speaking countries, from whom acceptances have been received.

A very large number of delegates from American States have been named and accepted their appointments.

The management congratulates itself and the Congress that the month of October was selected for the session of the Congress, and takes pleasure in announcing that all indications point to a large and successful session.

The management have organized a symposium on "Preventive Legislation Against Tuberculosis," and a letter has been sent out to those who take an interest in the work.

Standing committees will have charge of the following subjects: State Sanatoria, the Pathology and Bacteriology of Tuberculosis, Climatology, the Surgery of Tuberculosis, Light and Electricity, the usual standing committees on Resolutions, Ways and Means, Censorship of Papers, the Public Press, etc.

The Medico-Legal Society of New York has been invited to sit in joint session with the Congress, and it has accepted the invitation, and the floor of the Congress and its work is open to every member of that body and its sections.

[Judge Clark Bell, of New York, Chairman Committee on Organization, will have a strong paper in our next issue.—DANIEL.]

*First Vice-President of the Congress*—Dr. F. E. Daniel, President State Medical Association of Texas, Austin.

*Honorary Vice-Presidents of the Congress*—Prof. John T. Moore, M. D., Vice-President State Medical Association of Texas, Galveston; Dr. Geo. R. Tabor, State Health Officer of Texas; Dr.

T. J. Bennett, Ausin, Texas; Hon. Jos. D. Sayers, ex-Governor of Texas.

*Delegates*—Dr. M. M. Smith, Secretary State Board Medical Examiners; member of the Tuberculosis Congress Committee of Censors, Austin, Texas; Dr. H. .K Leake, Dallas; Dr. J. J. Robert, Hillsboro; Dr. J. E. Gilcreest, Gainesville; Prof. J. F. Y. Paine, M. D., Medical Department University of Texas, Galveston; Prof. J. W. McLaughlin, M. D., Medical Department University of Texas, Galveston; Dr. W. H. Allen, Marlin, Texas; Dr. E. S. Cox, Galveston, Texas; Dr. M. B. Grace, Seguin, Texas; Dr. James Lovett, Liberty, Texas; Dr. J. D. Jordan, Madisonville, Texas; Dr. Paul M. Raysor, Bryan, Texas; Dr. G. M. Abney, Franklin, Texas; Dr. E. E. Guinn, Rusk, Texas; Dr. P. L. Campbell, Dallas, Texas; Dr. J. H. Alexander, Meridian, Texas; Dr. J. F. Edwards, Denton, Texas; Dr. G. B. Foscue, Waco, Texas; Dr. H. W. Cummings, Hearne, Texas; Dr. B. M. Worsham, Superintendent State Insane Asylum, Austin; Dr. Marvin L. Graves, Superintendent State Insane Asylum, San Antonio; Dr. Jno. S. Turner, Superintendent State Insane Asylum, Terrell; Dr. John Preston, Superintendent Epileptic Colony, Abilene; Dr. C. H. Wilkison, San Antonio; Prof. Bacon Saunders, M. D., Dean Medical College, Fort Worth; Dr. Irvin Pope, Tyler; Dr. P. C. Coleman, Colorado City; Dr. Geo. H. Lee, Galveston, Dr. W. G. Jameson, Chief Surgeon I. & G. N. R. R., Palestine; Dr. A. C. Scott, Chief Surgeon G., C. & S. F. R. R., Temple; Dr. A. C. Smith, Chief Surgeon Cotton Belt Railroad, Tyler; Dr. R. W. Knox, Chief Surgeon S. P. System, Houston; Dr. S. C. Red, Chief Surgeon H. & T. C. R. R., Houston;; Dr. W. A. Duringer, Chief Surgeon ————, Fort Worth; Dr. A. Garwood, New Braunfels; Dr. J. A. Rawlings, El Paso; Dr. D. R. Fly, Amarillo; Dr. W. E. Fowler, Prison Physician, Huntsville; Dr. Boyd Cornick, San-Angelo; Dr. W. R. Blaylock, McGregor; Dr. B. F. Kingsley, San Antonio.

